# Gitelman Syndrome in a Child Presenting With Polyuria and Polydipsia: Diagnostic Challenges in a Resource‐Limited Setting

**DOI:** 10.1155/crin/8879176

**Published:** 2026-05-31

**Authors:** Erneus Ernest, Devis Simbila

**Affiliations:** ^1^ Department of Paediatrics and Child Health, Mbeya College of Health and Allied Sciences, University of Dar es Salaam, Mbeya, Tanzania, udsm.ac.tz; ^2^ Department of Paediatrics and Child Health, Mbeya Zonal Referral Hospital, Mbeya, Tanzania, mzrh.go.tz

**Keywords:** Gitelman syndrome, hypokalemia, hypomagnesemia, polyuria, renal salt wasting tubulopathy

## Abstract

Gitelman syndrome (GS) is a rare inherited renal salt‐wasting tubulopathy characterized by hypokalemia, hypomagnesemia, and hypocalciuria. Its nonspecific presentation often overlaps with that of more common pediatric conditions, leading to delayed diagnosis, particularly in resource‐limited settings. We report an 11‐year‐old boy who presented with progressive weight loss, polyuria, polydipsia, and salt craving. His course was complicated by recurrent episodes of severe hypokalemia and hypomagnesemia, and he was initially evaluated for diabetes mellitus, other endocrine disorders, chronic infections such as tuberculosis, and malnutrition without a definitive diagnosis. During the index admission, he developed acute worsening of muscle weakness associated with severe hypokalemia and hypomagnesemia and biochemical findings consistent with renal salt wasting, supporting a diagnosis of GS in the absence of genetic testing. Management with correction of hypovolemia, electrolyte supplementation, liberal salt intake, and nutritional support led to marked clinical improvement and stabilization of biochemical abnormalities on follow‐up. This case highlights the importance of maintaining a high index of suspicion for GS in children presenting with polyuria, polydipsia, salt craving, and unexplained electrolyte disturbances, particularly in resource‐limited settings.

## 1. Introduction

Inherited hypokalemic salt‐losing tubulopathies are a group of disorders characterized by impaired renal sodium chloride (NaCl) reabsorption, resulting in inappropriate urinary salt loss and chronic volume depletion. These conditions arise from pathogenic variants in genes encoding tubular ion transport proteins and include Bartter syndrome (BS) and Gitelman syndrome (GS).

The reported prevalence of GS is estimated at approximately 1 in 40,000, making it among the common inherited renal tubulopathies; however, the true prevalence is likely higher due to underdiagnosis and incidental detection of asymptomatic individuals [1–3]. GS differs from BS primarily in the site of the tubular defect, with GS resulting from impaired sodium–chloride reabsorption in the distal convoluted tubule, whereas BS is caused by defects affecting sodium–chloride transport in the cortical and medullary thick ascending limb of the loop of Henle. The two conditions also differ in age of presentation: GS typically presents in adolescence or adulthood [1], while most forms of BS present in the neonatal period, except for BS type III, which may present later in childhood [4].

The diagnosis of GS is based on a combination of clinical presentation and characteristic biochemical abnormalities. Clinical features at presentation are often nonspecific and may include polyuria, polydipsia, salt craving, muscle weakness, growth failure, and weight loss [1]. Owing to their nonspecific nature, these symptoms may complicate early recognition and contribute to delayed diagnosis, with patients frequently undergoing evaluation for more common conditions such as diabetes mellitus, chronic malnutrition, endocrine disorders, or chronic infections before the correct diagnosis is established.

Biochemically, GS is characterized by chronic hypokalemia, hypomagnesemia, hypocalciuria, elevated renin levels, normal or mildly elevated aldosterone concentrations, and evidence of renal salt wasting, often accompanied by metabolic alkalosis. While genetic testing remains the gold standard for diagnostic confirmation, in many resource‐limited settings, the diagnosis relies on careful clinical assessment, exclusion of alternative causes, and recognition of the characteristic biochemical pattern.

We report a case of GS in an 11‐year‐old boy presenting with polyuria, polydipsia, and progressive weight loss, illustrating the diagnostic challenges of this condition in resource‐limited settings.

## 2. Case Presentation

**FIGURE 1:**
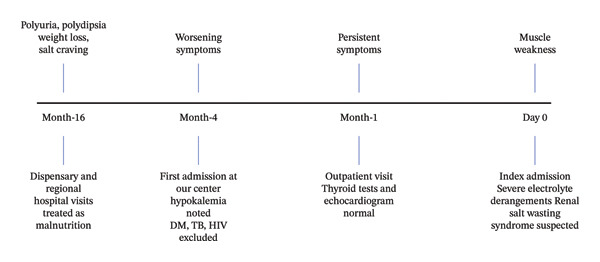
Timeline summarizing symptom onset, healthcare encounters, and key diagnostic milestones leading to the diagnosis of Gitelman syndrome. Negative values represent months prior to the index admission, and Day 0 denotes the index admission.

An 11‐year‐old boy was admitted with progressive muscle weakness that had acutely worsened over seven days, on a background of approximately 16 months of progressive weight loss, polyuria, polydipsia, and salt craving. During the early course of his illness, he attended several dispensaries and a regional hospital, where he was diagnosed with chronic malnutrition and received nutritional counseling without clinical improvement. Traditional remedies were also used without benefit. Details of laboratory investigations from these earlier visits were unavailable. There was no known family history of similar symptoms or parental consanguinity. The diagnostic journey from symptom onset to the index admission is summarized in Figure 1.

Approximately 4 months before the index admission, he was admitted to our center with persistent weight loss, polyuria, and polydipsia. On examination, he appeared wasted with features of volume depletion. Initial laboratory investigations revealed hypokalemia, while glycemic evaluation, renal function, and infectious workup were unremarkable. Despite these findings, no unifying diagnosis was established, and he was managed with intravenous fluids and potassium replacement before discharge.

During subsequent outpatient follow‐up approximately 1 month before the index admission, the patient continued to experience polyuria, polydipsia, salt craving, and progressive weight loss. Further evaluation, including thyroid function tests, chest radiography for tuberculosis screening, and echocardiography to assess cardiac causes of chronic illness, was unremarkable. Abdominal computed tomography, undertaken to evaluate for structural renal causes, showed mild dilatation of the right renal pelvicalyceal system and ureter, with no evidence of nephrolithiasis or obstruction. Despite these evaluations, a definitive diagnosis remained elusive.

Two months later, during the index admission, he re‐presented with worsening muscle weakness, progressing to difficulty walking and inability to ambulate independently. On examination, he had signs of mild chronic volume depletion, including dry mucous membranes and mild tachycardia, with normal blood pressure for age. His weight was 21 kg and height was 135 cm, corresponding to a body mass index of 11.6 kg/m^2^, indicating severe wasting and moderate stunting. The remainder of the systemic and neurological examination was unremarkable.

Biochemical evaluation revealed severe hypokalemia, hypomagnesemia, and hypocalcemia, with a normal serum sodium level. Given the recurrent electrolyte abnormalities and clinical features suggestive of renal salt wasting, a renal tubular disorder was suspected. Extended biochemical investigations demonstrated hypocalciuria and markedly elevated plasma renin levels, with inappropriate urinary chloride excretion despite clinical evidence of volume depletion. Taken together, these findings were consistent with GS. Detailed laboratory values during admission, discharge, and follow‐up are summarized in Table 1.

**TABLE 1:** Serial laboratory results during admission, discharge, and follow‐up.

Investigation	Previous admission	Outpatient visit	Index admission	At discharge	One‐month follow‐up	Reference range
Potassium (mmol/L)	2.3	—	1.9	3.4	3.1	3.5–5.1
Sodium (mmol/L)	—	—	136	134	134	136–145
Chloride (mmol/L)	—	—	95	95	—	98–107
Calcium (mmol/L)	—	—	1.53	2.03	2.30	2.15–2.55
Magnesium (mmol/L)	—	—	0.40	1.34	0.60	0.70–0.86
Creatinine (μmol/L)	40	27	35	—	—	62–106
Urea (mmol/L)	3.8	4.0	—	—	—	0–8.3
Fasting glucose (mmol/L)	4.8	—	4.79	—	—	3.9–5.5
HbA1c (%)	5.1	—	4.72	—	—	< 5.7
Urinalysis	Normal	—	Normal	—	Normal	—
TSH (mIU/L)	—	0.28	0.45	—	—	0.5–5.0
Serum cortisol (ng/mL)	—	—	120	—	—	50–250
Plasma renin (ng/L)	—	—	818.4	—	—	2.7–27.7
Plasma aldosterone (pmol/L)	—	—	296	—	—	70–1066
Aldosterone–renin ratio	—	—	0.4	—	—	Reference range not available
Urine osmolality (mOsm/kg H2O)	—	—	333	—	—	300–900
24‐h urine calcium (mmol/24 h)	—	—	0.54	—	—	2.5–7.5
24‐h urine chloride (mmol/24 h)	—	—	213	—	—	100–300

*Note:* Other investigations, including liver enzymes, HIV serology, sputum GeneXpert, urinalysis, autoimmune markers, inflammatory markers, and hematologic indices, were unremarkable and are not shown. Reference ranges are based on the performing laboratory standards. HbA1c, glycated hemoglobin.

Abbreviation: TSH, thyroid‐stimulating hormone.

### 2.1. Differential Diagnosis

Bartter syndrome (BS) was considered, given the presence of hypokalemia and secondary hyperreninemia. However, it was considered less likely for several reasons. First, most BS subtypes present antenatally or in the neonatal period, whereas later childhood presentation is uncommon except for BS type III [4]. Second, BS is typically associated with hypercalciuria and early nephrocalcinosis [5]. Although urinary calcium excretion may be normal or mildly reduced in type III BS, our patient demonstrated marked hypocalciuria (24‐hour urinary calcium excretion of 0.54 mmol/24 h), which is a characteristic feature of GS. Third, our patient demonstrated marked hypomagnesemia due to renal magnesium wasting, which is a characteristic and consistent feature of GS. In contrast, magnesium handling is usually normal in most BS subtypes and only variably affected in types III and IV [1, 3, 6]. Taken together with the later childhood age of presentation, these findings favor GS over BS in this patient.

Type 1 diabetes mellitus was considered due to the patient’s young age and the presence of polyuria, polydipsia, and weight loss. However, it was excluded for several reasons. First, repeated laboratory evaluations demonstrated normal fasting blood glucose and normal glycated hemoglobin, excluding sustained hyperglycemia. Second, despite a prolonged clinical course, the patient did not develop clinical or laboratory features suggestive of diabetic ketoacidosis, such as hyperglycemia or ketonuria. Taken together, these findings make diabetes mellitus unlikely in this patient.

Diabetes insipidus was considered due to the presence of polyuria and polydipsia. However, it was excluded for several reasons. First, diabetes insipidus is characterized by a water diuresis rather than renal salt wasting and is typically associated with normal or elevated serum sodium concentrations. In this patient, serum sodium levels remained within the normal range, and there was persistent hypokalemia and hypomagnesemia, which are not features of diabetes insipidus. Second, the presence of hypovolemia in the absence of hypernatremia further argued against diabetes insipidus. Taken together, these findings make diabetes insipidus unlikely in this patient.

### 2.2. Final Diagnosis

#### 2.2.1. Clinically Diagnosed GS

The diagnosis of GS was established based on a characteristic clinical and biochemical profile in the absence of genetic confirmation. Current consensus guidance (KDIGO) outlines a set of diagnostic features for GS, including chronic hypokalemia with inappropriate renal potassium wasting, metabolic alkalosis, hypomagnesemia, hypocalciuria, elevated renin levels, and normal or low blood pressure, together with exclusion of alternative causes [1].

In this case, the patient demonstrated persistent hypokalemia, hypomagnesemia, hypocalciuria, elevated plasma renin levels, and evidence of renal salt wasting, all consistent with these diagnostic features. The clinical presentation of polyuria, polydipsia, salt craving, and chronic volume depletion further supported the diagnosis.

Alternative causes of hypokalemia, including BS, diabetes mellitus, and diabetes insipidus, were systematically excluded based on age at presentation, electrolyte profile, and laboratory findings. Although metabolic alkalosis could not be confirmed due to the unavailability of arterial blood gas analysis, the overall clinical and biochemical constellation is consistent with accepted diagnostic features of GS, particularly in resource‐limited settings.

#### 2.2.2. Management and Outcome

During the index admission, the patient was managed with intravenous fluid resuscitation to correct hypovolemia, along with targeted correction of electrolyte abnormalities. Intravenous potassium chloride was administered at a dose of 0.5 mmol/kg (10 mmol diluted) under cardiac monitoring. Given the presence of hypocalcemia and hypomagnesemia, intravenous magnesium sulfate was administered first (1000 mg diluted in 5% dextrose over 3 h), followed by intravenous calcium gluconate at a dose of 0.5 mL/kg of 10% solution, diluted 1:1 with 5% dextrose and infused slowly over 20 min.

Following stabilization, long‐term therapy aimed at reducing renal salt wasting and maintaining electrolyte balance was initiated. Dietary sodium supplementation was prescribed at two teaspoons of sodium chloride per day, divided across meals (equivalent to approximately 210 mEq/day of sodium chloride). The patient and caregivers were counseled on increasing intake of locally available potassium‐rich foods, including ripe bananas, avocados, potatoes, and yams.

Oral potassium chloride (8 mEq per tablet) was initiated at one tablet every 12 h, providing approximately 0.8 mEq/kg/day. Oral magnesium aspartate was initiated at a dose providing approximately 0.15 mmol/kg/day of elemental magnesium based on the patient’s body weight and available tablet strength. Nutritional counseling was provided to ensure adequate caloric intake and support catch‐up growth.

This resulted in clinical improvement with stabilization of electrolyte abnormalities, although not fully normalized at discharge. The patient was discharged with close outpatient follow‐up.

At the 1‐month follow‐up visit, the patient demonstrated clinical improvement. Generalized weakness had reduced significantly, and he was able to ambulate better compared with the index admission. Symptoms of polyuria and polydipsia had also decreased and were no longer severely interfering with his daily activities. No significant adverse effects related to potassium or magnesium supplementation were reported, and adherence to therapy was reported as good during follow‐up.

Follow‐up biochemical testing demonstrated improvement in electrolyte abnormalities, with mild declines in potassium and magnesium compared with discharge values following transition to oral therapy, consistent with ongoing renal losses (Table 1).

Given that serum potassium remained above 3.0 mmol/L and magnesium was at the lower acceptable target range, no dose adjustment was made at that time, in keeping with recommended treatment targets and the need to balance biochemical correction with tolerability.

Financial constraints limited further investigations, and the patient was scheduled for continued follow‐up with another visit planned 1 month later to guide ongoing treatment adjustment.

## 3. Discussion

Although Gitelman syndrome (GS) is the most common inherited renal salt‐wasting tubulopathy [1], its diagnosis is frequently delayed due to nonspecific clinical features. Symptoms such as polyuria and polydipsia overlap with more common pediatric conditions, including diabetes mellitus and diabetes insipidus. In sub‐Saharan Africa, progressive weight loss often prompts evaluation for conditions such as tuberculosis and human immunodeficiency virus (HIV) infection, which may further delay consideration of a renal tubular disorder. This challenge is compounded in resource‐limited settings where access to genetic testing for confirmation of GS is limited. Consequently, timely diagnosis relies on a high index of suspicion and careful integration of clinical features with characteristic biochemical abnormalities, as illustrated in the present case.

GS is an autosomal recessive disorder caused by pathogenic variants in the *SLC12A3* gene, which encodes the thiazide‐sensitive sodium–chloride cotransporter (NCCT) located on the apical membrane of distal convoluted tubule epithelial cells [7]. To date, more than 350 *SLC12A3* mutations have been described [8]. NCCT mediates sodium and chloride reabsorption in the distal convoluted tubule; its dysfunction results in increased distal sodium delivery, chronic volume depletion, and subsequent activation of the renin–angiotensin–aldosterone system (RAAS). Aldosterone‐mediated sodium reabsorption in the collecting duct is accompanied by enhanced potassium secretion, accounting for the persistent hypokalemia characteristic of GS as demonstrated in this patient.

Hypomagnesemia is another cardinal biochemical feature of GS. Magnesium reabsorption in the kidney occurs predominantly in the proximal part of the distal convoluted tubule via the transient receptor potential melastatin 6 (TRPM6) channel. This nephron segment is highly dependent on NCCT activity for sodium transport; therefore, chronic NCCT dysfunction leads not only to functional impairment but also to abnormal development and structural remodeling, including segmental atrophy, of the distal convoluted tubule [9]. These changes are accompanied by reduced expression of the TRPM6 channel, resulting in impaired magnesium reabsorption and the profound renal magnesium wasting observed in patients with GS [10].

In GS, there is reduced renal excretion of calcium, although the underlying mechanism remains incompletely understood and is likely multifactorial. Chronic sodium chloride loss leads to mild volume depletion, prompting compensatory increases in sodium reabsorption in the proximal convoluted tubule, with calcium following passively [10, 11]. This mechanism contributes to hypocalciuria and explains the relative absence of nephrocalcinosis in GS. In addition, distal convoluted tubule adaptation to NCCT dysfunction by increased activity or expression of distal calcium transport pathways is thought to further enhance calcium reabsorption and sustain hypocalciuria independent of volume status [9]. These mechanisms are consistent with the markedly reduced 24‐hour urinary calcium excretion observed in our patient.

Patients with GS maintain normal serum sodium concentrations despite chronic renal salt wasting. This is explained by impaired sodium and chloride reabsorption in the distal convoluted tubule, which leads to increased distal sodium delivery and salt‐driven diuresis with polyuria. The resulting mild extracellular volume depletion activates the RAAS, promoting distal sodium reabsorption at the expense of increased potassium excretion. Through these compensatory mechanisms, serum sodium levels are preserved despite ongoing salt wasting, while hypokalemia persists. Increased dietary salt intake, driven by salt craving as seen in our patient, further contributes to the preservation of sodium homeostasis, consistent with the normal serum sodium concentrations observed in this case.

Consistent with this pathophysiology, urinary indices in our patient supported ongoing renal salt wasting. Urinary chloride excretion was not suppressed despite clinical evidence of volume depletion, indicating inappropriate renal sodium chloride loss. Taken together with persistent hypokalemia, hypomagnesemia, hypocalciuria, and elevated plasma renin levels, these findings were consistent with GS.

Our patient presented with muscle weakness and fatigue, features that have been well described in GS. Chronic hypokalemia and hypomagnesemia impair neuromuscular excitability and muscle contractility, leading to generalized weakness and fatigue [1, 12]. Our patient also had weight loss and was stunted. Growth failure has been reported in pediatric patients with GS and is attributed to chronic biochemical derangements and, in some cases, disruption of the growth hormone–insulin–like growth factor axis [13, 14].

Diagnosis of GS relies on a characteristic clinical and biochemical pattern rather than on isolated hormone measurements, and biochemical features may show considerable variability [1]. In this patient, aldosterone levels were within the normal range despite biochemical and clinical evidence of renal salt wasting. Aldosterone secretion in GS may vary and is influenced by volume status, dietary sodium intake, posture, and timing of sampling. Blood sampling was performed in the supine position due to severe muscle weakness, which increases effective circulating volume and suppresses aldosterone secretion, while frequent salt ingestion may have further attenuated aldosterone elevation. The presence of elevated renin, persistent hypokalemia, and clinical hypovolemia nonetheless supports ongoing RAAS activation in this patient.

Computed tomography of the abdomen demonstrated mild dilatation of the right pelvicalyceal system and ureter, with no evidence of nephrocalcinosis, renal calculi, or urinary tract obstruction. The patient had no clinical features of nephrolithiasis or voiding difficulty, and renal function remained normal. In the absence of obstructive pathology, this finding was considered incidental and may reflect functional dilatation related to chronic polyuria and impaired urinary concentrating ability associated with renal salt wasting.

There is no curative therapy for GS; management is supportive and lifelong, aiming to correct electrolyte abnormalities, alleviate symptoms, and prevent complications. Treatment is centered on liberal dietary sodium intake, potassium chloride supplementation, and magnesium replacement, with correction of hypomagnesemia being essential to facilitate potassium repletion. Potassium supplementation is titrated with a target serum potassium level of at least 3 mmol/L, recognizing that complete normalization may not be achievable in all patients. Similarly, magnesium targets are individualized, with levels around 0.6 mmol/L often considered an acceptable lower threshold, balancing biochemical improvement with tolerability [1]. Nutritional support is important to address weight loss and growth failure. Ongoing follow‐up is required to monitor clinical response, electrolyte levels, renal function, and treatment‐related adverse effects, particularly gastrointestinal intolerance to magnesium therapy. Additional pharmacologic therapies, such as nonsteroidal anti‐inflammatory drugs or potassium‐sparing agents, are not routinely indicated in GS and were therefore not used in this patient [1, 3].

This case adds to the growing body of literature highlighting the diagnostic challenges of GS due to its nonspecific and overlapping clinical presentation. Similar to previous reports, including that by Melkie et al. [15], patients may experience delayed recognition due to nonspecific symptoms. This case underscores how symptom overlap can contribute to delayed diagnosis, particularly in resource‐limited settings where access to specialized investigations is limited. Notably, this case illustrates a prolonged diagnostic course in a child initially managed for malnutrition and other common conditions, emphasizing the need for earlier consideration of renal tubulopathies in similar clinical contexts. It emphasizes the importance of maintaining a high index of suspicion and integrating clinical and biochemical findings to facilitate earlier recognition of this condition. A structured clinical approach based on recognition of characteristic biochemical patterns and systematic exclusion of alternative causes is essential, particularly in settings where genetic testing is not readily available.

This case has some limitations. First, arterial blood gas analysis was not performed due to a lack of availability in our setting; therefore, metabolic alkalosis, a typical biochemical feature of GS, could not be formally documented. Second, confirmatory genetic testing was not available, precluding molecular confirmation of the diagnosis. Nonetheless, the diagnosis was supported by a characteristic clinical presentation, consistent biochemical abnormalities, exclusion of alternative causes, and a favorable response to targeted therapy. Third, the duration of follow‐up was relatively short at the time of manuscript preparation, limiting assessment of long‐term outcomes; however, follow‐up is ongoing.

## 4. Conclusion

GS is a rare and underrecognized renal tubular disorder, particularly in children, in whom its nonspecific symptoms often overlap with more common conditions, leading to delayed diagnosis. Diagnostic evaluation is further challenged in resource‐limited settings by financial constraints and lack of access to confirmatory genetic testing. This case highlights the importance of maintaining a high index of suspicion and relying on a characteristic clinical and biochemical pattern rather than isolated laboratory values. Early recognition and appropriate supportive management can result in meaningful clinical improvement and prevent avoidable morbidity.

## Author Contributions

Erneus Ernest contributed to patient evaluation, diagnosis, and management and drafted the initial manuscript. Devis Simbila contributed to patient evaluation and ongoing clinical care and coordinated the acquisition of specialized laboratory investigations, in addition to maintaining close follow‐up with the patient and caregivers. All authors provided input during manuscript development.

## Funding

No funding was received for this work.

## Disclosure

All authors approved the final version for submission.

## Ethics Statement

Ethical approval was not required for this study as it describes a single patient. According to the guidelines of the authors’ institution, case reports involving a single patient do not require formal ethics committee approval. Written informed consent was obtained from the patient’s legal guardian for publication of this report, including all relevant clinical information and images.

## Consent

Written informed consent was obtained from the patient’s legal guardian (father) for publication of this case report.

## Conflicts of Interest

The authors declare no conflicts of interest.

## Data Availability

Data sharing is not applicable to this article as no datasets were generated or analyzed during the current study.
